# Inhaled nitric oxide stabilizes acute right ventricular failure due to hypercapnia in abdominal compartment syndrome: a case report

**DOI:** 10.1186/s40981-026-00857-5

**Published:** 2026-04-14

**Authors:** Yusuke Takei, Yuna Sato, Hidehisa Saito, Hirona Nishimaki, Shoma Tanaka, Hiroaki Toyama, Yutaka Ejima, Masanori Yamauchi

**Affiliations:** 1 Department of Anesthesiology and Perioperative Medicine, Tohoku University Graduate School of Medicine, 2-1 Seiryo-machi, Aoba-ku, Sendai, Miyagi-ken 980-8574 Japan; 2 Department of Anesthesiology, Osaki Citizen Hospital, Osaki, Japan; 3 Department of Anesthesiology, Yamagata University Faculty of Medicine, Yamagata, Japan

**Keywords:** Abdominal compartment syndrome, Right ventricular failure, Pulmonary hypertension, Hypercapnia, Inhaled nitric oxide

## Abstract

**Background:**

Inhaled nitric oxide is a selective pulmonary vasodilator commonly used to treat pulmonary hypertension and right ventricular failure. However, its effectiveness in treating right ventricular failure secondary to hypercapnia caused by abdominal compartment syndrome has not been well described.

**Case presentation:**

This case describes a patient who developed severe hypercapnia and acute right ventricular failure due to abdominal compartment syndrome following massive intraoperative hemorrhage. Elevated intra-abdominal pressure impaired the respiratory mechanics, resulting in alveolar hypoventilation, hypercapnia, and acute pulmonary hypertension. Respiratory and circulatory failures progressively worsened. Administration of inhaled nitric oxide was associated with a rapid improvement in right ventricular afterload surrogates, leading to improved right ventricular output and enhanced carbon dioxide elimination, with concomitant improvement in systemic hemodynamics.

**Discussion:**

Inhaled nitric oxide may serve as an effective rescue therapy for hypercapnia-induced pulmonary hypertension and acute right ventricular failure associated with abdominal compartment syndrome.

**Supplementary Information:**

The online version contains supplementary material available at 10.1186/s40981-026-00857-5.

## Background

An acute increase in pulmonary vascular resistance (PVR) can precipitate right ventricular (RV) failure, particularly in conditions associated with alveolar hypoventilation and severe hypercapnia [1]. Abdominal compartment syndrome (ACS), characterized by sustained intra-abdominal hypertension, impairs respiratory mechanics and may lead to hypoventilation, acute pulmonary hypertension, and RV overload [2].

Inhaled nitric oxide (iNO) is a selective pulmonary vasodilator that reduces pulmonary vascular resistance, thereby improving ventilation–perfusion matching without causing systemic hypotension [3]. Although commonly used for pulmonary hypertension, its role in acute RV failure secondary to hypoventilation-induced PVR elevation in ACS is not well established.

We report a case of massive intraoperative hemorrhage complicated by ACS in which iNO administration resulted in rapid hemodynamic stabilization, suggesting a potential role as rescue therapy in this setting.

## Case presentation

A man in his 60s underwent extended right hepatectomy for perihilar cholangiocarcinoma. During parenchymal transection, injury to the middle and left hepatic veins caused massive bleeding (estimated blood loss, 52,323 mL). After intraperitoneal gauze packing and temporary hemostasis, the abdomen was closed with plans for postoperative coagulopathy correction.

In the intensive care unit, persistent bloody drainage (> 200 mL/h) from a subdiaphragmatic drain required continued transfusion of packed red blood cells and fresh frozen plasma. Normothermia was maintained. After partial correction of coagulopathy, the drain was clamped to increase intra-abdominal pressure (IAP) and promote tamponade of venous bleeding; the intravesical pressure at that time was 7 mmHg.

Over the following hours, the abdomen became tense and distended, accompanied by oliguria and progressive deterioration of respiratory mechanics, consistent with evolving ACS. As IAP rose to 58 mmHg, the patient became anuric and developed severe metabolic acidosis requiring continuous renal replacement therapy (CRRT). IAP was measured using the standard intravesical technique via a urinary catheter. Measurements were obtained in the supine position after instillation of 25 mL of saline into the bladder, with the pressure transducer zeroed at the level of the mid-axillary line at end expiration. The patient was deeply sedated and mechanically ventilated at the time of measurement.

Paroxysmal atrial fibrillation occurred with hypotension, and although cardioversion restored sinus rhythm, hemodynamic instability persisted. Ongoing transfusion and fluid resuscitation led to pulmonary edema consistent with transfusion-associated circulatory overload.

**Fig. 1:**
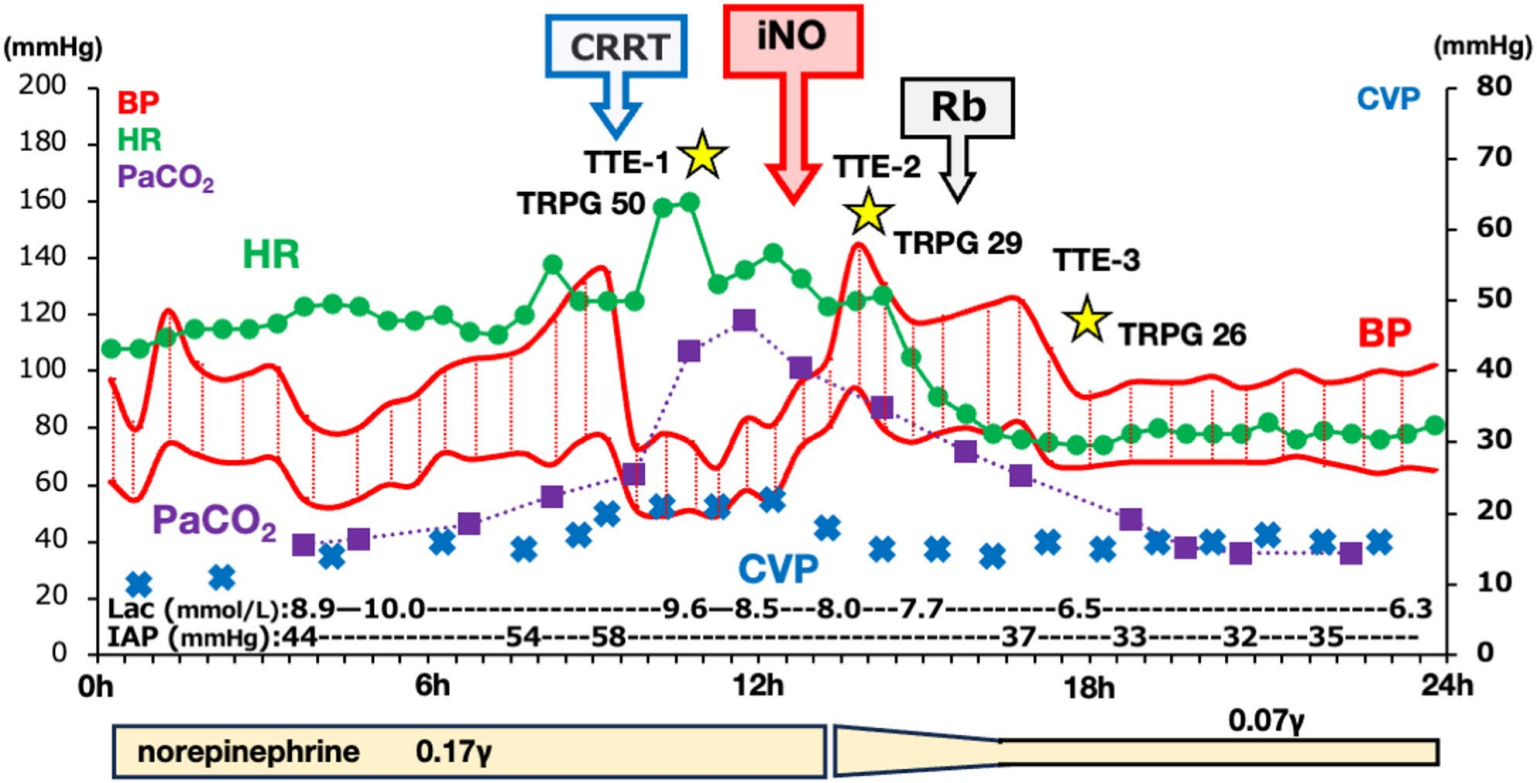
Hemodynamic and respiratory parameters before and after inhaled nitric oxide (iNO) administration. Time-course changes in key hemodynamic and respiratory parameters before and after iNO initiation. Following iNO therapy, marked improvements in hemodynamic parameters (CVP, BP) and PaCO2 were observed, consistent with reduced right ventricular afterload, despite minimal change in minute ventilation.TRPG, tricuspid regurgitation pressure gradient; iNO, inhaled nitric oxide; TTE, transthoracic echocardiography; IAP, intra-abdominal pressure; BP, blood pressure; CVP, central venous pressure; Rb, rocuronium bromide; CRRT, continuous renal replacement therapy.

Pulmonary congestion and elevated IAP markedly impaired lung compliance. Despite pressure-controlled ventilation (peak inspiratory pressure 32 cmH₂O, PEEP 5 cmH₂O, FiO₂ 1.0), tidal volume remained < 200 mL. Arterial blood gas analysis showed a PaO₂/FiO₂ ratio of 129 and PaCO₂ of 118 mmHg, indicating severe hypoxemic and hypercapnic respiratory failure. Further escalation of catecholamines was avoided because of severe lactic acidosis. Hemodynamic and respiratory parameters before and after subsequent interventions are summarized in Table 1; Fig. 1.

**Table 1:** Changes in hemodynamic and respiratory parameters following inhaled nitric oxide and rocuronium administration

	Before iNO treatment	60 min after iNO treatment	After Rb administration	After surgical decompression
P/F ratio	129	211	280	173
PaCO_2_ (mmHg)	118	87.2	63.4	46.9
EtCO_2_ (mmHg)	53	55	47	37
PaCO_2_ –EtCO_2_ gap (mmHg)	65	32.2	16.4	9.9
Vd/Vt (Enghoff approximation)	0.55	0.369	0.259	0.211
RR (/min)	24	24	20	15
Tidal volume (mL)	163	171	340	453
MV (L/min)	3.9	4.1	6.8	6.8
BP (mmHg)	66/49 (55)	144/94 (111)	98/68 (78)	118/61 (99)
HR (/min)	158	121	68	78
CVP (mmHg)	22	15	16	11
RV/LV ratio	1.49	1.01	1.03	NM
TRPG (mmHg)	50	30	26	NM
IAP (mmHg)	58	NM	37	17
NED (µg/kg/min)	0.169	0.169	0.102	0.037
Lac (mmol/L)	9.6	8.0	6.5	3.4

IAP at 60 min after iNO initiation and echocardiographic parameters (RV/LV ratio and TRPG) after surgical decompression were not measured because clinical management prioritized hemodynamic stabilization

*iNO* inhaled nitric oxide, *Rb* rocuronium bromide, *BP *blood pressure, *HR* heart rate, *CVP* central venous pressure, *P/F* ratio, PaO_2_/FiO_2_ ratio, *PaCO*₂0 arterial partial pressure of carbon dioxide, *EtCO*₂ end-tidal carbon dioxide, *Vd/Vt* physiological dead space fraction (Enghoff approximation), *RR* respiratory rate, *MV* minute ventilation, *RV* right ventricle, *LV* left ventricle, *TRPG* tricuspid regurgitation pressure gradient, *IAP* intra-abdominal pressure, *NED* norepinephrine equivalent dose, *Lac,* lactate, *NM* not measured

**Fig. 2:**
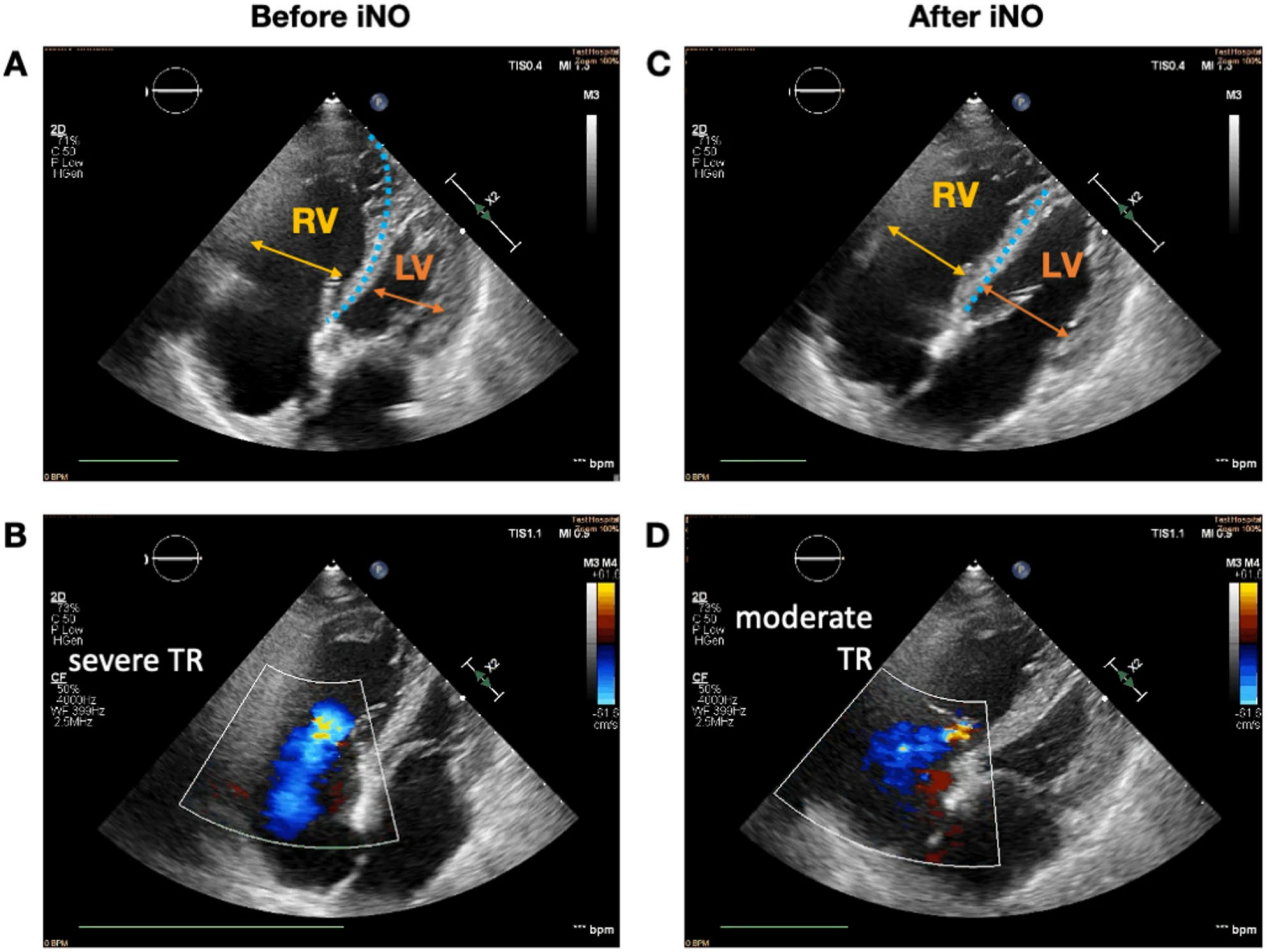
Transthoracic echocardiography findings before and after inhaled nitric oxide (iNO) administration. **A** Apical four-chamber view before iNO administration showing marked RV dilation (RV/LV > 1.0) and leftward deviation of the interventricular septum, consistent with acute RV pressure overload. **B** Color Doppler image before iNO administration showing severe tricuspid regurgitation with a TRPG of 50 mmHg. **C** Apical four-chamber view after iNO administration showing reduction in RV dilation and improved interventricular septal position and RV/LV ratio, demonstrating reduced RV afterload. **D** Color Doppler image after iNO administration showing attenuation of tricuspid regurgitation with a TRPG of 30 mmHg. Abbreviations: RV, right ventricle; LV, left ventricle; TRPG, tricuspid regurgitation pressure gradient; iNO, inhaled nitric oxide.

Transthoracic echocardiography (TTE) revealed acute RV pressure overload, including RV dilation (basal RV/left ventricular ratio > 1.0), and an elevated tricuspid regurgitation pressure gradient of 50 mmHg (Fig. 2) [4]. These findings were consistent with those of acute pulmonary hypertension secondary to severe hypercapnia and impaired ventilation associated with ACS. Pulmonary artery catheterization was not performed because of the patient’s profound hemodynamic instability and the urgent need for a noninvasive assessment.

Inhaled nitric oxide was administered at 20 ppm using the INOflo^®^ system with the INOflo^®^ DS delivery device and INOflo^®^ inhalation gas (800 ppm cylinder concentration; Mallinckrodt Pharmaceuticals, USA). The decision followed multidisciplinary discussion among the critical care and surgical teams. This off-label use was undertaken as an emergency rescue intervention based on clinical judgment in a rapidly deteriorating hemodynamic situation, and it was consistent with the institutional framework for off-label administration of inhaled nitric oxide at our hospital.

Within minutes, the tricuspid regurgitation pressure gradient decreased from 50 to 30 mmHg, central venous pressure fell from 22 to 14 mmHg, and systolic blood pressure increased from 66 to 96 mmHg, indicating rapid hemodynamic stabilization (Fig. 1). At 60 min after iNO initiation, respiratory rate remained unchanged (24/min), minute ventilation increased minimally (3.9 to 4.1 L/min), and tidal volume showed no substantial change (163 to 171 mL). Despite this, PaCO₂ decreased from 118 to 87.2 mmHg. The PaCO₂–EtCO₂ gradient decreased from 65 to 32.2 mmHg, and estimated Vd/Vt declined from 0.55 to 0.369, suggesting improved pulmonary perfusion rather than enhanced alveolar ventilation (Table 1). Methemoglobin levels remained within the normal range (1.4–1.5%).

Rocuronium bromide was administered after hemodynamic stabilization with iNO. Following neuromuscular blockade, IAP decreased from > 50 mmHg to 38 mmHg, accompanied by an increase in tidal volume and further improvement in hypercapnia. Echocardiographic parameters, including TRPG and the RV/LV ratio, suggested a reduction in the RV afterload and stabilization of the circulatory status. Re-exploration surgery was subsequently performed, and the ongoing venous bleeding was successfully controlled. After definitive hemostasis and surgical decompression, systemic hemodynamics remained stable with a marked reduction in serum lactate levels. Urine output remained negligible, and renal replacement therapy was continued (Table 1).

Following decompression and continued intensive care management, hemodynamic and respiratory parameters transiently stabilized. However, during his prolonged intensive care unit stay, the patient developed Enterococcus faecium bacteremia. Serum β-D-glucan levels were elevated, raising suspicion of concomitant invasive fungal infection. Antimicrobial and antifungal therapy was initiated. Despite treatment, progressive hepatic failure developed, and the patient died from septic complications one month after definitive hemostatic surgery.

## Discussion

This case illustrates a pathophysiological cascade in which ACS caused alveolar hypoventilation and severe hypercapnia, leading to elevated PVR and acute RV failure. Although hypercapnia likely played a dominant role in increasing PVR, hypoxemia and volume overload may also have contributed to RV dysfunction. Decompressive laparotomy is the definitive treatment for ACS; however, this patient was too hemodynamically unstable to tolerate abrupt IAP reduction [5]. iNO was administered as a physiological bridge before definitive surgery. Notably, PaCO₂ decreased after iNO administration despite unchanged minute ventilation.

Transthoracic echocardiography demonstrated acute RV pressure overload with RV dilation, septal flattening, and elevated TRPG. After iNO administration, RV geometry improved, TRPG decreased, systemic arterial pressure increased, and central venous pressure fell, consistent with reduced RV afterload and recovery of RV output. Although pulmonary artery catheterization was not performed, the hemodynamic changes strongly suggested PVR reduction. iNO was selected because it selectively lowers PVR without worsening systemic hypotension.

Hypercapnia is a recognized cause of pulmonary vasoconstriction and pulmonary hypertension. Prior studies have shown that iNO can attenuate hypercapnia-associated increases in pulmonary arterial pressure [6].

However, these reports have largely focused on pulmonary hemodynamics and gas exchange rather than on the reversal of overt RV failure. Clinical evidence on the use of iNO specifically for hypercapnia-induced pulmonary hypertension, which is severe enough to precipitate acute RV failure, remains limited. In our patient, hypercapnia-related elevation of PVR occurred in the setting of markedly reduced venous return due to ACS, resulting in a critical imbalance between the RV preload and afterload, and subsequently led to circulatory collapse.

The marked reduction in PaCO₂ following iNO administration, despite minimal changes in ventilator settings and minute ventilation, suggests improved pulmonary perfusion rather than enhanced alveolar ventilation. During the first 60 min after iNO initiation, the PaCO₂–EtCO₂ gradient decreased by more than 50%, and estimated Vd/Vt declined from 0.55 to 0.369. No major concurrent changes in vasopressor dose, ventilatory parameters, or fluid management occurred during this period (Fig. 1), supporting a temporal association with iNO. Although minute ventilation increased slightly, the magnitude of PaCO₂ reduction exceeded what would be expected from this small change alone. Because regions with reduced perfusion function as physiological dead space, improved pulmonary blood flow through relief of RV afterload likely played a dominant role in enhancing carbon dioxide elimination [7]. Experimental data suggest that such improvements are closely linked to increases in cardiac output and redistribution of pulmonary blood flow [8].

At extremely elevated IAP, abdominal vascular compression reduces effective venous return by minimizing unstressed volume. Sudden decompression or neuromuscular relaxation under these conditions may abruptly increase vascular capacitance, promote venous pooling, and precipitate circulatory collapse [9]. In this patient, elevated IAP was intentionally maintained for hemostasis, making decompression particularly high risk. Optimization of RV function and systemic hemodynamics was therefore essential before neuromuscular blockade and laparotomy.

This case does not advocate routine off-label use of iNO. Rather, iNO served as a temporary physiological bridge to stabilize RV failure and gas exchange until definitive management of ACS was achieved. The patient ultimately died from infectious complications after prolonged critical illness, underscoring that iNO should be regarded as a rescue strategy rather than a disease-modifying therapy.

This report has limitations. PVR was inferred from echocardiographic and hemodynamic findings rather than measured directly, and dead-space ventilation was estimated rather than quantified. In addition, iNO was administered off-label, which limits generalizability.

In conclusion, severe ACS can precipitate acute RV failure through hypercapnia-induced pulmonary hypertension. In selected life-threatening situations, iNO may function as a physiological bridge by reducing RV afterload, improving RV output, and facilitating CO₂ elimination, thereby enabling safer surgical decompression. This case emphasizes the value of individualized physiological assessment and targeted cardiopulmonary support in complex perioperative ACS. 

## Supplementary Information

Supplementary Material 1.

## Data Availability

All data generated or analyzed during this study are included in this published article.

## Abbreviations

RV: Right ventricle
LV: Left ventricle
TRPG: Tricuspid regurgitation pressure gradient
iNO: Inhaled nitric oxide
