# Supplementary material for: Inhaled nitric oxide stabilizes acute right ventricular failure due to hypercapnia in abdominal compartment syndrome: a case report
Source: JA Clin Rep. 2026 Apr 14;12:23. doi: 10.1186/s40981-026-00857-5 (PMC13187105; doi:10.1186/s40981-026-00857-5)
Supplement: Supplementary file 1 — Supplementary Material 1. [file 40981_2026_857_MOESM1_ESM.docx]

**Response to Reviewers**

**Response to Reviewer #1**

Thank you very much for your insightful comments.

**1. Abstract—wording regarding RV afterload**

We appreciate your suggestion. As this is a single case report and pulmonary vascular resistance was not directly measured, we have revised the wording to avoid implying causality.

The sentence has been modified to:

“was associated with a rapid improvement in right ventricular afterload surrogates”

**2. Figure 1 legend—minute ventilation**

Thank you for pointing this out. We have corrected the legend to accurately reflect the data:

“despite minimal change in minute ventilation”

**3. Table 1—unit for minute ventilation**

We have corrected the unit as suggested:

MV (L/min)

**Response to Reviewer #2**

We sincerely thank the reviewer for the careful evaluation of our manuscript and for confirming that the revisions were appropriate.

We believe that these revisions have addressed the reviewers’ comments and improved the clarity of the manuscript. We sincerely appreciate the reviewers’ valuable suggestions.
